# Observations on the Blood in Asthma, Epilepsy, Hydro-Pericardium, and Bilious Fever

**Published:** 1846-09

**Authors:** J. R. Buck

**Affiliations:** Louisville, Kentucky


Art. III.—*Observations on the Blood in Asthma, Epilepsy, Hydro-pericardium, and Bilious Fever.* By J. R. Buck, M.D., of Louisville, Kentucky.

The physical changes in the blood as to color, consistence, coagulability, the formation of the cup, or the buffy coat, are inconstant,—occurring in diseases opposite in character, and of little or no diagnostic value. We are compelled, therefore, to seek in those intimate chemical changes in the blood, the nature of all diseases in which this fluid is either primarily or secondarily involved. Marshall Hall practises bloodletting as a diagnostic, as well as therapeutic measure; tolerance by the system of the loss of a large amount of blood indicating inflammation, the want of this tolerance, the absence of inflammation. But to practise this means of diagnosis, even if it could be relied upon, would require the abstraction of an amount of blood injurious to the patients in all affections not inflammatory, whereas in the process of analysing that fluid the results, even in the present state of our knowledge of its pathology, afford us far more certain information, and the quantity necessary to be taken is very small.

I have examined the blood in phthisis pulmonalis, pneumonia, scrofula, chronic rheumatism, hydro-pericardium, epilepsy, asthma, and bilious fever. Reports of the four last mentioned cases only will be given in the present communication. In some future number I may publish the result of the observations upon the blood in phthisis, showing a wide difference in its chemical constitution in the different stages of the disease. The method which I pursued in these analyses was similar to that of Andral, Gavarret, and Zimmerman, except in the process of obtaining the blood corpuscles which, I believe, is more accurate, at the same time that it is much less complicated, than the process pursued by these experimenters.

Case I.—R. S., æt. 76 years, a farmer, born and raised on the Eastern shore of Maryland, of temperate habits, and with no family predisposition to disease of any kind, he enjoyed uninterrupted health until quite an old man. At 71 years of age he had some difficulty in discharging his urine; this increased for several months when retention became complete, and the catheter had to be used; since which time, nearly five years, the bladder has never been evacuated without the use of this instrument. At 73 he was occasionally troubled with palpitation, dyspnœa, and slight cough; the dyspnœa increased for about a year, the attacks becoming more frequent, and more severe, while the palpitation lessened; the feet and ancles, in the meantime, beginning to swell. In January, 1843, I first saw him; the dyspnœa was then constant, and, upon taking the least exercise, very intense; attended with a sensation of tightness across the chest; countenance expressive of great anxiety; cough loose, with mucous expectoration; pulse irregular, full; impulse of the heart scarcely perceptible, sounds feeble but natural in character; great prominence in the precordial region.

*Diagnosis.*—Hydro-pericardium. Prescription—four cut cups to the region of the heart; cream of tartar and jalap. This treatment caused marked improvement in a few days. Calomel, squills, and digitalis were then given, and subsequently syrup of the iodide of iron. Under this treatment the patient seemed to recover perfectly in a few months; has had since, at intervals of several months, attacks of dyspnœa, with violent palpitation. Bleeding from the arm, and leeching have been practised, but not with the same good effects which followed the application of cups.

On the 29th of June, I was called to see him; he had been suffering for several days with dyspnœa, and a sensation of stricture as though a cord was drawn tightly around the chest. Pulse was full and strong, but could be compressed; dry cough; slight cephalalgia. Took from the arm about nine ounces of blood, which yielded upon analysis:—

| | |
|---|---|
| Water - - - - - - | 798 |
| Solid residue - - - - - | 202 |
| Fibrin - - - - - - | 2·3 |
| Albumen - - - - - - | 65·4 |
| Blood corpuscles, - - - - | 122 |
| Salts and extractive matter - - | 12·6 |

It will be seen from this analysis, that the fibrin, (an increase of which is said by Andral always to indicate inflammatory action,) is not at all above the healthy standard; it is indeed rather below the amount, assumed by some writers as the average, in the healthy adult. The albumen and blood corpuscles are also rather below the healthy standard. The relative amount of water to the solid matters varies but little from the healthy average.

What practical deductions are we to draw from this observation? That the blood was healthy, all the constituents being within the range of healthy blood; that, whatever may have been the nature of this disease in the first stage, there existed no inflammatory action at the time the observation was made; but the fullness of the pulse, pain of the head, &c., depended upon some other morbid condition. The day after making this analysis I prescribed the counter-irritating lotion of Granville with very decided benefit; and upon a generous diet, with gentle tonics, the patient has continued to improve.

Case II.—A merchant, æt. 27 years, of healthy parentage, in the summer of 1845 had a slight attack of asthma, which passed off in a few days without treatment. In January, 1846, he had a severe attack of tonsillitis confining him to his bed eight days, from which he recovered entirely in a short time after getting up. In the latter part of June he was taken in the night with a severe attack of asthma. I saw him next morning when his condition was as follows: Respiration exceedingly laborious, with a feeling of oppression as though a heavy weight was confined upon the chest; cough almost constant, dry and not deep; pulse 92, full and of moderate firmness; percussion elicited no unnatural sound; respiratory sounds feeble, with an occasional loud, intensely rough inspiration. Bled from arm to the extent of twelve ounces, when the patient became quite faint; the dyspnœa was somewhat relieved by the bloodletting; five hours after an emetic of tartrate of antimony and ipecacuan was administered, which relieved the paroxysm. Since that time he has had one very slight attack, for which he was not subjected to treatment.

ANALYSIS OF THE BLOOD.

| | |
|---|---|
| Temperature - - - - - | 98° |
| Specific gravity - - - - | 1032 |
| Fibrin - - - - - - - | 1·3 |
| Albumen - - - - - | 66·8 |
| Blood corpuscles - - - - | 77·1 |
| Salts and extractive matter - - | 10 |
| Water - - - - - - | 843 |
| Solid residue, - - - - - | 157 |

The amount of solids to the water is much less in this case than in the first, and is below the healthy standard. 211 may be assumed as the average of solid residue in one thousand parts of healthy blood; 190 and 270 being the extremes. The fibrin is diminished to about one-half the normal proportion. The blood corpuscles are diminished in the same ratio as the fibrin. The albumen and salts are within the healthy range. There was nothing unusual in the blood as it ran from the arm; it was neither very pale, nor very dark; it coagulated readily and formed a buffy coat, but was not cupped.

By comparing this analysis with that of the blood in inflammatory diseases, a directly opposite condition will be found in the quantity of fibrin. In a case of pneumonia involving extensively both lungs, as was proved by a post-mortem examination, the fibrin had increased to 6·5 in the thousand parts of blood.

Case III.—This was a case of congenital epilepsy, in a bright mulatto, æt. 25 years. This patient has a dull, heavy look, and a whining voice, but seems otherwise to be perfectly healthy; he was raised on a farm in Arkansas and always accustomed to lead an active life. The fits occur about once a month, but at no particular phasis of the moon, and have varied in this respect but little since the patient can recollect. He does not know that the disease was referred by his mother to any particular cause; is not aware of any family predisposition to disease of any kind. At the time the blood was taken for analysis he seemed to be in good health; his appetite was good, bowels regular, pulse and respiration natural. The following was the character of the blood:

| | |
|---|---|
| Temperature - - - - - | 96° |
| Specific gravity - - - - | 1031 |
| Water - - - - - - | 805 |
| Solid residue - - - - - | 195 |
| Fibrin - - - - - - | 2 |
| Blood corpuscles - - - - | 129·8 |
| Salts and extractive matter - - | 10·1 |
| Albumen - - - - - | 55 |

The blood in this case cannot be regarded as in a pathological condition. The albumen is rather below the average, but is within the healthy range.

All three of these cases come under the head of the neuroses; for although in case first inflammation doubtless existed, in the earlier stages of the disease, it had been subdued many months before the blood was examined; a strictly nervous condition existing at the time. I have called it hydro-pericardium because this was the original disease, and probably the exciting cause of the nervous affection. There was no increase in the constituents of the blood in either of these cases above the healthy range; in one, a very marked decrease in the two most important elements, the fibrin and blood corpuscles.

Case IV.—The subject of the next case was æt. 20 years; generally healthy; joined the Kentucky cavalry about the 1st of June, and encamped in a flat and rather low situation, badly watered; was here exposed to much rain and intensely hot weather, a greater portion of the tine without tents; his food being pickled pork and bean soup.

On the 28th of June he was taken with a severe chill followed by fever; next day the chill returned. I saw him on the 4th of July; he had had no chill since the 29th of the month before, but constant fever, with slight remissions; had taken Cooke's pills, blue pills, quinine, and brandy toddy; was then (afternoon of the 4th) lying in a half stupid condition; upon being aroused would talk hurriedly, but was rational. His breathing was laborious, with an uneasy sensation in the chest; respiratory sounds more intense than natural; pulse 91, full and compressible; very considerable deafness; other senses perfect. Tongue covered in the centre with a whitish fur; very slight tenderness upon pressure in the epigastric, and right and left hypochondric regions; skin inclined to be hot. Has had two discharges from his bowels during the morning, which were thin and light colored.—Blood to the amount of twelve ounces was taken from the arm, followed next day by twelve leeches to the anterior portion of the chest. The breathing was somewhat improved by the depletion. Prescribed nitrous powders every two hours until perspiration was produced; next day quinine and opium, 20 grains of the former, to 1 grain of the latter were given at a single dose, and repeated every morning for three days, when the fever began to yield. Three days subsequent to discontinuing the quinine and opium profuse watery discharges from the bowels came on, which had to be restrained by opium, acetate of lead, and a large blister over the abdomen.

July 12. Patient convalescent. I subjoin a partial analysis of the blood in this case, its completion having been defeated by the mistake of a medical friend and assistant.

| | |
|---|---|
| Temperature - - - - - | 100° |
| Specific gravity - - - - | 1028 |
| Fibrin, - - - - - - | .5 |

It is much to be regretted that this analysis was not perfected. The temperature was higher, by several degrees, than I have found it in chronic pneumonia and chronic rheumatism, in which the fibrin was nearly double the healthy standard, and yet in this specimen the fibrin is very greatly diminished.

July 27, 1846.

